# Genotype characteristics of two non traveler sources *burkholderia pseudomallei* strains isolated from clinical patients for the first time in Chongqing, China

**DOI:** 10.1186/s12879-025-11819-0

**Published:** 2025-10-14

**Authors:** Gang Duan, Chunyan Liao, Gaomin Liu, Linghan Kong, Ling Wang

**Affiliations:** 1 Chongqing Center for Disease Control and Prevention, Microbial Testing Institute, Chongqing, 400707 China; 2 Chongqing Academy of Preventive Medical Sciences, Chongqing, 400707 China; 3 Chongqing Municipal Key Laboratory for High Pathogenic Microbes, Chongqing, 400707 China; 4 Chongqing wanzhou District Center for Disease Control and Prevention, Chongqing, 404100 China

## Abstract

In 2024, two cases of melioidosis were discovered in Chongqing, China. This was the first instance of non-traveler infection cases being found in this region. The strain characteristics and phylogenetic relationships of the two *B. pseudomallei* (CQ2024Bp-001 and CQ2024Bp-002) remained poorly understood. An antibiotic sensitivity test, genomic sequencing, prediction of virulence genes and ARGs, wgSNP, and MLST were performed for the two strains. The results indicated that the two strains had significant genetic differences (17197 SNPs), and CQ2024Bp-002 was a novel sequence type (ST2149). CQ2024Bp-001 carried *bla*_OXA-59_ and *bla*_OXA-57_, while CQ2024Bp-002 only carried *bla*_OXA-59_. Despite carrying $\beta$-lactam resistance genes, both strains were sensitive to common antibiotics (TIM, CAZ, MEM, MH, LEV, SXT, and CPL). Their virulence genes differed, with CQ2024Bp-002 lacking *boaB* and *wzt2*. The clustering analysis based on wgSNP revealed that the two strains were distant from each other. These findings suggest that under the influence of global warming, non-endemic areas of melioidosis are gradually developing conditions suitable for the reproduction and transmission of *B. pseudomallei*, potentially leading to an increase in melioidosis cases. It highlights the need for an active monitoring system to be established in climate-sensitive areas to prevent and control the potential regional epidemic of melioidosis.

## Introduction

*Burkholderia pseudomallei* (*B.pseudomallei*) is a Gram-negative partially intracellular pathogen that causes Melioidosis, an anthropozoonosis. Studies have shown that *B. pseudomallei* primarily exists in the water and soil of endemic areas. It infects humans via three primary routes: the respiratory tract, the digestive tract, and broken skin. This infection causes melioidosis, an acute or chronic infectious disease with a wide spectrum of clinical manifestations. Mild cases often present with non-specific initial symptoms such as fever, fatigue, and cough, while severe cases can progress to life-threatening complications, including sepsis, severe pneumonia, and multiple abscesses that commonly involve the liver, lungs, or subcutaneous tissues. the incubation period is usually 1–21 days [1, 2]. Melioidosis was first reported in 1911 in Rangoon, Myanmar. It is mainly endemic in tropical and subtropical countries and regions between 20° south and north latitudes, with most cases reported in Southeast Asia and northern Australia [3]. In China, it is predominantly distributed in Hainan, Guangdong, Guangxi, HongKong, Fujian, and Taiwan [4–6].

Due to global warming, non-traveler cases in non-endemic areas have been reported in recent years. Limmathurotsakul D et al. estimated the global disease burden of melioidosis based on known human, animal cases and modeling of environmental melioidosis strains. Their estimation suggests that the disease is grossly underreported in 45 countries where it is known to be endemic, and melioidosis may be endemic in 34 countries where the disease has never been reported [7]. With its variable symptoms, high misdiagnosis rate, high disease risk and mortality rate of 10–40%, melioidosis is a growing public health problem [8, 9].

Chongqing city is located at near 30°N latitude and in the subtropical region, which is not a traditional source of melioidosis, and cases of melioidosis are rare. In the past 10 years, only one imported case from Thailand in 2017 [10] and one imported case from Guangdong, China in 2021 were documented in the literature [11]. No local infection cases were reported. However, in June and July 2024, *B. pseudomallei* was isolated from blood samples of 2 male patients aged 60 years or older. Epidemiologic investigation revealed that one patient never left Chongqing within one year, while the other patient did not leave Chongqing city one month prior to the onset date. There was no overlap in their activity trajectories, and both were urban residents without farming experience. Neither had been exposed to foreign imports, which initially suggested two independent cases of local infection.

What were the genomic characteristics and antibiotic resistance of the two strains, and why were there two consecutive cases of melioidosis in Chongqing in 2024? We studied the antibiotic sensitivity, virulence genes, genome sequencing and cluster traceability analysis of the two strains to explore the genotypic characteristics, pathogenicity and antibiotic resistance of the two local *B. pseudomallei* strains found for the first time in Chongqing, China.

## Methods

### Source of strains

Suspected strain isolated from the blood of 2 patients (strain ID: CQ2024Bp-001; CQ2024Bp-002)

### Instruments and main reagents

MALDI-TOF MS(Bruker, Germany), Real time fluorescence quantitative PCR instrument(BORI Technology, China), nucleic acid extractor (Bioperfectus Technologies, China), biosafety cabinet (ESCO, Singapore), Qubit fluorescence quantitative instrument (Thermo Fisher, USA), MGISP100 automatic library builder(MGI, China), DNBSEQ G99 s-generation sequencer (MGI, China), Columbia blood plate (Dijing, China), Gram-negative bacteria antibiotic sensitivity plate and broth medium CAMHB (ThermoFisher, USA), bacterial DNA nucleic acid extraction kit (BioperfectusTechnologies, China), *B.pseudomallei* fluorescence PCR detection kit(MABSKY, China), and sequencing-related reagents andconsumables (MGI, China). All reagents were used within the validity period.

### Strain culture and identification

Single colonies were picked and inoculated in Columbia blood plates at 37 °C for 18 h-24 h for pure culture, and fresh pure cultures were taken for identification. After picking single colonies and inactivating the bacteria by formic acid method, Matrix-Assisted Laser Desorption/Ionization Time of Flight Mass Spectrometry(MALDI-TOF MS) was used to identify the strains. MBT compass software was used to compare and analyze the whole-cell protein fingerprints of the bacteria to be tested for rapid identification. Species identification of strains using commercialized fluorescent PCR kits, The target gene is BPSS1386, which encoding ATP-binding protein of *B. pseudomallei*. Bacterial DNA was extracted by boiling method, and the reaction system was configured and amplification parameters were set according to the instructions of the kit.

### Genome sequencing

DNA of two strains was extracted using the Bacterial DNA Nucleic Acid Extraction Kit, Geneme sequencing was performed after constructing libraries using the Enzymatic DNA Library Preparation Kit (Paired-end sequencing,100bp per read). FastP 0.23.1 (https://github.com/OpenGene/fastp) was used for the quality control of raw data [12], kraken2 2.1.2(https://github.com/-DerrickWood/kraken2) was used for the identification of bacterial species [13], and draft genomes were assembled in SPAdes 3.5.0 (https://github.com/ablab/spades.git) software [14].

### Antibacterial susceptibility assays and Prediction of Antibiotic resistance genes(ARGs)

Took single colony into broth medium, adjustd the turbidity to 0.5 McFarland units, and used the microbroth dilution method to detect the minimum inhibitory concentration (MIC) of the strains to commonly used antibiotics, including the following antibiotics: Ticarcillin/clavulanic acid(TIM), Ceftazidime(CAZ), Meropenem(MEM), Minocycline(MH), Levofloxacin(LEV), Trimethoprim/sulfamethoxazole(SXT), Chloramphenicol(CPL). The quality control strain was Escherichia coli ATCC25922. The interpretation of antibiotic sensitivity test results was based on the sensitivity or resistance standards for *burkholderia cepacia Complex* in the 2023 edition of the Clinical Laboratory Standards Institute(CLSI) [15]. ResFinder4.6.0 (http://genepi.food.dtu.dk/resfinder) was used to predict acquired antimicrobial resistance genes,parameter settings:Threshold for lD:90.0%. Minimum length:60.0% [16].

### Prediction of virulence genes

The virulence factor analysis was Performed by Virulence Factors of Pathogenic Bacteria(VFDB) (http://www.mgc.ac.cn/VFs/) [17].

### MLST typing and clustering analysis

MLST was performed by using genome assembly to query the *B.pseudomallei* MLST database (https://pubmlst.org/organisms-/burkholderia pseudomallei) [18], The 7 housekeeping genes(*ace*, *gltB*, *gmhD*, *lepA*, *lipA*, *narK*, *ndh*) of *B.pseudomallei* were compared to obtain the ST type of the two strains. Then, we downloaded 64 genome sequences of *B.pseudomallei* which separated from different countries and regions since 1986 from National Center for Biotechnology Information (NCBI) (https://www.ncbi.nlm.nih.gov/datasets/). After determining the ST type of them, the MLST clustering was performed by GrapeTree (https://github.com/achtman-lab/GrapeTree/releases), NJ and MSTree V2 minimum spanning tree were used to generate the grapetree [19].

### Clustering analysis based on wgSNPs

The genome sequences of the two strains and other 64 strains downloaded from NCBI were analyzed for single nucleotide polymorphisms (wgsnps).The evolutionary tree was constructed based on the maximum likelihood method using the open source software: Fasttree, bootstap requirements more than 70%. Cross-genus transmission events was illustrated using the online server of iTOL [20]. The template files for iTOL were generated using itol.toolkit [21].

## Results

### Strains identification and Colony morphology

Two strains were cultured on the Columbia blood agar plates for 24 h and there were small, smooth, moist, protruding, transparent or semi-transparent colonies with complete borders. After 48 h of culture, a dry, opaque white colony was formed, resembling a “wheel-like”. Gram staining of these bacteria was negative, and microscopic colony morphology was ovoid in shape. The MALDI-TOF MS identification results were *B. pseudomallei*, Log(score):2.29;the fluorescent PCR test results were *B. pseudomallei* too.

### Genome sequencing results

Strain identification using kraken2 confirmed that both strains were *B.pseudomallei*.the CQ2024Bp-001 genome included 151 contigs,with a size of 7.1 Mb,and a GC content of 68.2%,5872 CDS,5952 gene,2 rRNA,77 tRNA,1 tmRNA; the CQ2024Bp-002 genome included 137contigs, with a genome size of 7.2 Mb, and a GC content of 68.03%,5940 CDS,6022 gene,2 rRNA,79 tRNA,1 tmRNA. the genome sequence differences between the two strains were 23916 SNPs.

### Results of antibiotic susceptibility test and ARGs

According to the interpretation of CLSI M45,CQ2024Bp-001 and CQ2024Bp-002 were both sensitive to TIM,CAZ,MEM,MH, LEV,SXT, and CPL. Resistance gene analysis revealed that CQ2024Bp-001 carries the $\beta$-lactam antibiotic resistance genes *bla*OXA-59 and *bla*OXA-57. CQ2024Bp-002 carries the $\beta$-lactam antibiotic resistance gene *bla*OXA-59 (Table 1).

**Table 1:** Antibiotic susceptibility test results and ARGs carrier of the two *B. pseudomallei* strains

		CQ2024Bp-001		CQ2024Bp-002	
Antibiotic	Interpretive categories	MIC ($\mu$ g/mL)	ARGs	MIC ($\mu$ g/mL)	ARGs
TIM	S $\le$16/2	$\le$16/2		$\le$16/2	
CAZ	S $\le$8	$\le$8		$\le$8	
MEM	S $\le$4	$\le$4	blaOXA-59	$\le$4	blaOXA-59
MH	S $\le$4	$\le$4	blaOXA-57	$\le$4	
LEV	S $\le$2	$\le$2		$\le$2	
SXT	S $\le$2/38	$\le$2/38		$\le$2/38	
CPL	S $\le$8	$\le$8		$\le$8	

*Abbreviations*: *MIC* minimum inhibitory concentration, *S* sensitive, *ARGs* antibiotic resistance genes

### MLST typing and cluster analysis

**Fig. 1:**
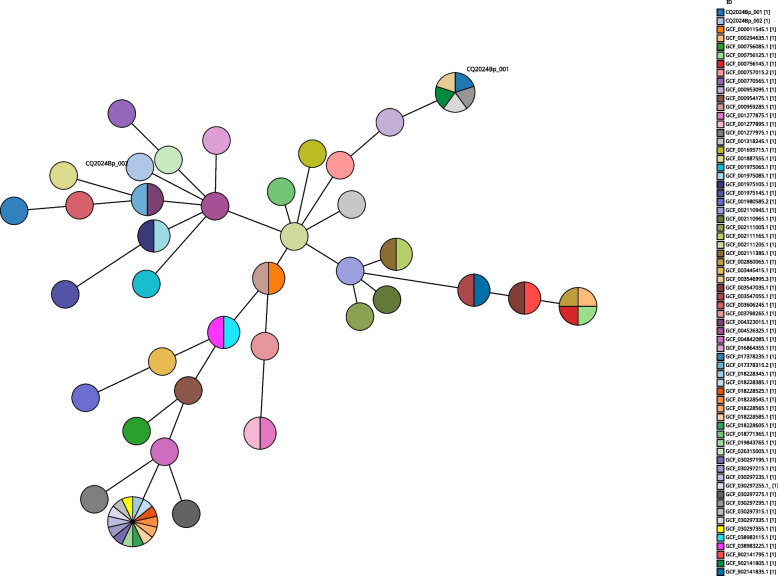
MLST cluster analysis of 66 *B.pseudomallei* genomes

The genome sequences of two strains were submitted to PubMLST, CQ2024Bp-001 was identified as ST46 and CQ2024Bp-002 was a new ST, ST2149 was assigned. 64 *B.pseudomallei* genome sequences were subjected to MLST typing, and MLST clustering analysis was carried out by using grapetree software. CQ2024Bp-001 and CQ2024Bp-002 were distributed in different clades, CQ2024Bp-001 was in the clade where GCF-004526325 (isolated from Sri Lanka) was located. However, CQ2024Bp-002 was in the clade where GCF-002111205 (isolated from California, USA) was located (Fig. 1).

### Prediction results of virulence genes

The results of the virulence gene analysis are shown in Table 2.

**Table 2:** Distribution of main virulence factors of *Burkholderia pseudomallei* genomes CQ2024Bp-001 and CQ2024Bp-002

Virulence factor	Related gene	Missing genes (CQ2024Bp-001)	Missing genes (CQ2024Bp-002)
BimA	bimA	N^a^	N
Type IV pili	pilA, pilB, pilC, pilD, pilN, pilO, pilQ, pilR, pilS, pilT, pilV	N	N
Adhesion protein	boaA, boaB	N	boaB
Capsule I	gmhA, manC, wcbA, wcbB, wcbC, wcbD, wcbE, wcbF, wcbG, wcbH, wcbI, wcbJ, wcbK, wcbL, wcbM, wcbN, wcbO, wcbP, wcbQ, wcbR, wcbS, wcbT, wzm, wzt2	N	wzt2
Flagella	cheA, cheB, cheD, cheR, cheW, cheY1, cheY, cheZ, flgA, flgB, flgC, flgD, flgE, flgF, flgG, flgH, flgI, flgJ, flgK, flgL, flgM, flgN, flhA, flhB, flhF, fliA, fliC, fliD, fliE, fliF, fliG, fliH, fliI, fliJ, fliK, fliL, fliM, fliN, fliO, fliP, fliQ, fliR, fliS, motA, motB, tsr	N	N
Bsa Type III Secretion System (Bsa T3SS)	bapA, bapB, bapC, basJ, bicA, bicP, bipB, bipC, bipD, bopA, bopC, bopE, bprA, bprB, bprC, bprD, bprP, bprQ, bsaK, bsaL, bsaM, bsaN, bsaO, bsaP, bsaQ, bsaR, bsaS, bsaT, bsaU, bsaV, bsaX, bsaY, bsaZ, orgA, orgB, spaP	N	N
Type VI Secretion System 1 (T6SS1)	clpV	N	N

^a^N means that this strain completely carries all the genes listed in the corresponding related genes column, and there is no deletion of any of the genes indicated in this column

### WgSNPs phylogenetic developmental tree construction

**Fig. 2:**
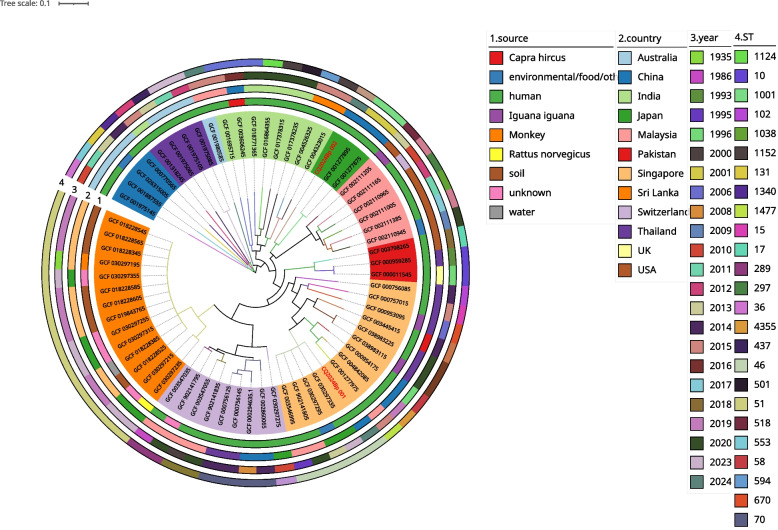
Phylogenetic of the 66 *B. pseudomallei* strains. Tree based on single-nucleotide polymorphisms(SNPs) identified by whole genome SNP-site among the strains. Different colours on the strains name show the clustering of strains. The accompanying heatmap visualises key strains characteristics, including isolation sources, country, year and ST type

The results of the maximum likelihood phylogenetic tree (Fig. 2) show that geographically, the two strains isolated in Chongqing are far away from the Australian strains in terms of evolutionary distance, and they were close to the Asian strains. the Australian strains are closer to the root of the tree. CQ2024Bp-001 and CQ2024Bp-002 are distributed in different clades and have a relatively distant relationship. Both strains are significantly different from the *B. pseudomallei* GCF-003606245 isolated from Hainan Province, an epidemic area of melioidosis in China. The closest relatives to CQ2024Bp-001 are GCF-030297335 isolated from a patient in Osaka, Japan, in 2023, GCF-030297295 isolated from the outer environment of Osaka, Japan, in 2013, GCF-902141805 isolated from a patient in Malaysia in 2020, GCF-003546995 and GCF-003546995 isolated from patients in Malaysia in 1995. Differently, most similar to CQ2024Bp-002 are GCF-001277895 and GCF-001277875 isolated from patients in Taiwan, China in 2001.

## Discussion

First, Combined with the two patients’ no travel history, no import exposure history and the genomic characteristics of the strains, it is speculated that *B.pseudomallei* may exist in the natural environment of Chongqing, but this conclusion needs to be supported by direct evidence. At present, the research is only based on clinical strains, lacking direct evidence of homologous strains isolated from soil, water and other environmental samples in Chongqing. In the future, more sufficient verification should be obtained through environmental sampling and culture to confirm the colonization status of the strain in the local environment. Genome sequencing and clustering analysis based on MLST and wgsnp showed: the two *B. pseudomallei* strains isolated for the first time in Chongqing differed significantly, and they were distantly related, which implied that they had different origins or transmission routes. In particular, the discovery of the new ST type 2149 has furthered our understanding of the genetic variation and evolutionary relationships of *B.pseudomallei*. As human-to-human transmission of melioidosis is rare, individuals exposed to environments containing *B. pseudomallei* were usually infected through inhalation or skin contact [21]. In this study, the two cases had not left Chongqing for more than 1 month, and the onset time was concentrated between June and July 2024. Although the incubation period of melioidosis may be very long from infection to the onset of clinical symptoms, Viseth Ngauy et al. reported a case in which the onset of disease occurred 62 years after the initial exposure [22]. However, Matthew Howes et al. retrospectively studied the historical report of melioidosis caused by the activation of the incubation period. It was believed that the vast majority of historical cases were undiagnosed chronic melioidosis, which was usually a relapse remission process, rather than a real latent and asymptomatic infection. Latency activation is a rare event in melioidosis, accounting for less than 3% of historical cases [23]. Christopher K et al. reported 4 cases of melioidosis due to the use of aromatic therapy spray exported from India to the United States contaminated by *B.pseudomallei* [24]. However, the two patients in Chongqing had not been exposed to any imported products. Due to the rare cases of melioidosis with a incubation period of several years or even decades, the short-term non travel history of the patients in this study is only one of the references to support local infection, not the only evidence. Subsequently, the possibility of latent period activated imported infection needs to be further ruled out through environmental strain isolation and genome comparison. In tropical areas, the prevalence of melioidosis is seasonal. The vast majority of cases occur in the rainy season. In addition to contact with *B. pseudomallei* contaminated soil and infected water, extreme weather may lead to a change in the mode of infection. Heavy rainfall produces aerosols carrying *B. pseudomallei*, which can infect human by inhalation or other means [25]. Currie, B.J reported that in Northern Australia, continuous rainstorms led to more melioidosis infected people with severe symptoms such as pneumonia. The highest mean rainfall(211 mm) was recorded in the 14 days prior to hospitalization of patients who died from melioidosis, and the rainfall in the 14 days before admission was an independent risk factor for melioidosis pneumonia, bacteremia, sepsis, shockand death [26]. Both cases in Chongqing denied having a history of contact with soil and epidemic water, the rainy season in Chongqing is from April to September every year. We queried the historical weather information of Chongqing [27]. The average precipitation reached 205 mm in June 2024. From June 19 to 21 and from June 26 to 28, Chongqing had strong convective and rainy weather, which had the weather conditions to form aerosol and cause human infection. The traditional endemic areas of *B. pseudomallei* are the Southeast Asian region and northern Australia. Pearson et al. concluded that *B. pseudomallei* originated in Australia and then moved to Southeast Asia, from where it spreaded to the rest of the world, based on the analysis of SNPs in the whole genome sequence [28]. The evolutionary tree we constructed also supported this conclusion, with the Australian strains appearing earlier, the two Chongqing strains evolving later and being most closely related to strains isolated in recent years from neighboring regions in Asia. In recent years, more and more cases which are not associated with travel had been identified in areas outside endemic regions [29–31]. In 2022, CDC reported *B. pseudomallei* isolated from the soil and water in the Gulf Coast region of Mississippi and two cases of melioidosis associated with the same strain of *B. pseudomallei*. This is the first time that *B. pseudomallei* has been identified in the environment in the continental US [32]. According to statistics from National Climate Centre(NCC) of China, the average temperature in China in 2024 was 10.9°C, which was 1.01°C higher than the usual(9.89°C), surpassing the 10.71°C in 2023 and setting a new historical high. The temperature in 19 provinces (cities, districts) including Chongqing was the highest since 1961 [33]. This suggests that due to changes in climate, the non-endemic areas of melioidosis are gradually becoming suitable for the reproduction and spread of *B. pseudomallei*, and there is a tendency toward increased infection in non-endemic areas. In Chongqing, a subtropical region, two consecutive cases of local melioidosis occurred in 2024, which also demonstrates that melioidosis has a trend of spreading in non-endemic areas, warranting increased attention and further research.

Secondly, the antibiotic of choice for melioidosis, CAZ, and the eradication phase drug, SXT, are both effective against the two strains. *B.pseudomallei* possesses natural resistance mechanisms to commonly used antibiotics such as quinolones, first and second generation cephalosporins, and penicillins. The two strains carry $\beta$-lactam antibiotic resistance gene *bla*OXA-59. The antibiotic sensitivity test results indicate that they are sensitive to quinolones such as LEV. Because clavulanic acid has a broad-spectrum $\beta$-lactamase inhibitory effect, TIM can protect ticarcillin from being hydrolyzed by $\beta$-lactamase and demonstrates bactericidal effects on both strains. SXT will be used increasingly in the treatment of melioidosis during the eradication phase [34]. Thai scholars Weerachai C et al. tested 769 strains isolated from patients in Laos and Cambodia for resistance and found only 5 strains were resistant to STX, concluding that resistance of *B.pseudomallei* to SXT is very rare [35]. The two strains are susceptible to SXT as well, which aligns with their findings.

Thirdly, the virulence genes carried by the two strains isolated from Chongqing differ, as CQ2024Bp-002 lacks virulence genes *boaB* and *wzt2*. *B.pseudomallei* possesses a vast bacterial genome (7.2M), encompassing a broad spectrum of virulence factors, which can cause various clinical manifestations including latent carrier state, acute and chronic pneumonia, local infection, sepsis and others [36]. The commonly identified virulence factors of *B.pseudomallei* include: flagella, which are essential for invading host cells. The flagellin subunit of *B.pseudomallei* is encoded by *fliC*(bpsl3319). The deletion of the *fliC* gene results in the emergence of non-motile strains [37]. The intracellular motility-related protein (*bimA*) evades the host immune mechanism. After adhering to host cells, *B.pseudomallei* secretes *bimA*, which induces the formation of actin filaments. These filaments help *B.pseudomallei* to move within and between cells, thereby evading phagocytosis by immune cells [38–40]. The type IV pili play a crucial role in adhesion, enabling the bacterium to infect multiple organs throughout the body. When *B.pseudomallei* lacks the special structural protein *pliA* of type IV pili, its adhesion to host cells is significantly reduced [41, 42]. The Bsa type III secretion system cluster 3(Bsa T3SS-3), T3SS-3 plays a pivotal role in the phagocytosis and escape of *B. pseudomallei* within host cells. Gram-negative bacteria are known to possess three sets of T3SS, among which only T3SS-3 is crucial in bacterial infection [43]. T3SS-3 is capable of secreting a unique effector protein: BopC. The inactivation of BopC leads to decreased bacterial survival within phagosomes [44, 45]. The capsular polysaccharide I(CPS) can protect *B.pseudomallei* from being phagocytosed by phagocytic cells [46]. The cluster I type VI secretion system(T6SS-1) is a crucial virulence factor for *B.pseudomallei* in cell-to-cell transmission, and it plays a pivotal role in facilitating the formation of multi-nucleated giant cells(MNGC) [47]. Both strains possess major virulence factors such as flagellum, Bsa T3SS-3, T6SS-1, BimA, Type IV pili and CPS. Additionally, key virulence genes such as *fliC*, *pliA*, and *bopC* are intact. However, CQ2024Bp-002 lacks the virulence genes *boaB* and *wzt2*. In previous studies, the boaB is involved in the regulation of specific virulence factors in some bacteria [48], and the wzt2 is related to the modification of lipopolysaccharides or capsular synthesis of bacteria, which can affect the pathogenicity of bacteria [49, 50]. However, this deletion phenomenon was only found at the gene level in this study. Because bacterial pathogenicity is a complex multifactorial process, changes in a single or few genes are not necessarily simply equivalent to changes in pathogenicity, but also involve the synergy of many other genes and the complex mechanism of bacterial host interaction. The lack of two genes is not sufficient to conclude that the pathogenicity of CQ2024Bp-002 is weaker. X.Y. Fu et al. analyzed the virulence factors of 52 strains of *B.pseudomallei* isolated from Hainan, China, and found significant differences in the genes of adhesion proteins *boaA* and *boaB*, with the *boaA* carrying rate of 86.54% and *boaB* carrying rate of only 7.69% [51]. The *boaA* and *boaB* genes of the two strains isolated from Chongqing align with this finding.

Deficiencies and follow-up studies: There are two limitations in this study: one is the lack of direct evidence of environmental strains in Chongqing, leading to incomplete evidence chain of ’local infection’ conclusion; Second, the sample size for strain comparison in China is insufficient, and the regional origin of the strains cannot be fully determined. Although the epidemiological history of the patients and the genomic characteristics of strains (the prevalence of St46 and the novelty of st2149) tend to be local infections, it cannot completely rule out import-related non-travel infections (such as indirect import through contaminated goods). We plan to download more genome data from representative strains in China, analyze and evaluate the distance matrix by comparing WgSNP and cgMLST, calculate the difference value of WgSNP and cgMLST, typing consistency between Chongqing strains and other Chinese strains, clarify their clustering position on the evolutionary tree of Chinese strains, and further judge the regional origin characteristics of St46 and st2149.

## Conclusion

In summary, the two cases of melioidosis discovered in Chongqing in 2024 are both non-traveler infections. The two strains of *B.pseudomallei* isolated demonstrate genetic diversity. This represents the first identification of local strains of *B.pseudomallei* in Chongqing, a non-endemic area of melioidosis. The route of infection for the two cases in Chongqing may be airborne.Although increasing Awareness, advancement in diagnostic facilities etc. may be factors for the first detection of local cases of melioidosis in Chongqing. Yet changes in temperature and humidity which caused by global warming is a important reason too. It may lead to the activation of *B.pseudomallei* in the environment of non-endemic areas such as Chongqing, increasing the risk of human or animal infection and potentially forming new epidemic areas. The two strains isolated in Chongqing are susceptible to antibiotics commonly used for melioidosis. This suggests that antibiotics such as CAZ could be considered as the antibiotic of choice if additional cases of melioidosis occur in Chongqing in the future. There are differences in virulence genes between the two strains. CQ2024Bp-002 lacks *boaB* and *wzt2*, suggesting that CQ2024Bp-002 has lower pathogenicity than CQ2024Bp-001. With global warming, more non-traveler cases of melioidosis may emerge in non-endemic areas. Public health departments should strengthen the monitoring of melioidosis, improve the early identification capabilities of medical staff, and guide residents in risk areas to avoid contact with contaminated soil and water sources.

## Data Availability

The sequence data for the 2 newly sequenced Chongqing B. pseudomallei isolates have been uploaded to the National Center for Biotechnology Information (NCBI). This project is registered as BioProject PRJNA1221073, the Sample numbers of B. pseudomallei CQ2024Bp-001 ST46 and CQ2024Bp-002 ST2149 are SAMN46726082 and SAMN46726083, respectively. The GenBank accession numbers are GCA-048159275.1 and GCA-048159185.1 for CQ2024Bp-001 and CQ2024Bp-002.

## Abbreviations

DNA: DeoxyriboNucleic Acid
PCR: Polymerase Chain Reaction
ARGs: Antibiotic resistance genes
MIC: Minimum inhibitory concentration
TIM: Ticarcillin/clavulanic acid
CAZ: Ceftazidime
MEM: Meropenem
MH: Minocycline
LEV: Levofloxacin
SXT: Trimethoprim/sulfamethoxazole
CPL: Chloramphenicol
ATCC: American Type Culture Collection
CLSI: Clinical Laboratory Standards Institute
MLST: Multi-Locus Sequence Typing
NCBI: National Center for Biotechnology Information
NJ: Neighbor-Joining
wgSNPs: Whole Genome Single Nucleotide Polymorphism
